# PDCD2 as a prognostic biomarker in glioma correlates with malignant phenotype

**DOI:** 10.1016/j.gendis.2023.101106

**Published:** 2023-09-21

**Authors:** Fengsheng Dai, Yixiao Yuan, Jiaqi Hao, Xing Cheng, Xiangyi Zhou, Li Zhou, Rui Tian, Yi Zhao, Tingxiu Xiang

**Affiliations:** a Department of Oncology, The First Affiliated Hospital of Chongqing Medical University, Chongqing 400010, China; b Chongqing Key Laboratory of Translational Research for Cancer Metastasis and Individualized Treatment, Chongqing University Cancer Hospital, Chongqing 400030, China; c Department of Neurosurgery, Xiangya Hospital, Central South University, Changsha, Hunan 410008, China; d Hunan International Scientific and Technological Cooperation Base of Brain Tumor Research, Xiangya Hospital, Central South University, Changsha, Hunan 410008, China; e Department of Neuro-Oncology, Chongqing University Cancer Hospital, Chongqing 400030, China

**Keywords:** Biomarker, Glioma, Programmed cell death 2, Progression, Solid cancer

## Abstract

Programmed cell death 2 (PDCD2) is related to cancer progression and chemotherapy sensitivity. The role of PDCD2 in solid cancers (excluding hematopoietic malignancies) and their diagnosis and prognosis remains unclear. The TCGA, CGGA, GEPIA, cBioPortal, and GTEx databases were analyzed for expression, prognostic value, and genetic modifications of PDCD2 in cancer patients. Functional enrichment analysis, CCK8, colony formation assay, transwell assay, and xenograft tumor model were undertaken to study the PDCD2's biological function in glioma (GBMLGG). The *PDCD2* gene was associated with solid cancer progression. In the functional enrichment analysis results, PDCD2 was shown to participate in several important GBMLGG biological processes. GBMLGG cells may be inhibited in their proliferation, migration, invasion, and xenograft tumor growth by knocking down *PDCD2*. Our research can provide new insights into solid cancer prognostic biomarkers of PDCD2.

## Introduction

Cancer is still a major disease threatening human health worldwide, bringing serious economic and medical burdens to society.^1^ Despite improvements in cancer diagnosis and treatment in recent years, some patients still have poor prognosis.^2^^,^^3^ The discovery of potentially reliable and characteristic biomarkers for cancer diagnosis and treatment is therefore crucial for improving cancer patient survival and prognosis.

Programmed cell death 2 (PDCD2) was first detected in rats,^4^ and is essential for the development of mice and fruit flies during embryonic development.^5^^,^^6^ In human cells, PDCD2 could participate in ribosome assembly as a ribosomal chaperone protein.^7^ PDCD2 has been shown to be associated with B-cell malignant tumors and is regarded as a target gene of BCL6, and patients with acute leukemia can be monitored by PDCD2.^8^, ^9^, ^10^ Emerging evidence has indicated that PDCD2 acts as a tumor suppressor in gastrointestinal stromal tumors,^11^ liver cancer,^12^ and gastric cancer.^13^ A sufficient understanding of PDCD2's role in carcinogenesis is still lacking, however.

Here, we examined the expression level, clinical characteristics, prognostic value, and mutation status of PDCD2 in solid cancers. PDCD2 was also evaluated on glioma (GBMLGG) cells using the CCK8, colony formation, transwell assays, and xenograft tumor model. Our findings indicate that PDCD2 could be used for cancer diagnosis and prognosis and as a molecular target for GBMLGG.

## Materials and methods

### Data acquisition and processing

Data from The Cancer Genome Atlas (TCGA) website (https://portal.gdc.cancer.gov/repository) and the Genotype-Tissue Expression (GTEx) database were applied.^14^ Furthermore, we obtained clinical and sequencing information from more than 2000 brain tumor samples sourced from the Chinese Glioma Genome Atlas (CGGA) website (http://www.cgga.org.cn/).^15^ Before analysis, the transcript per kilobase (TPM) values were standardized. Using the pROC and the ggplot2 package in R, the diagnostic value of PDCD2 was evaluated.

### UALCAN

Solid cancers PDCD2 protein expression levels were assessed using the UALCAN portal (http://ualcan.path.uab.edu),^16^^,^^17^ which is an interactive web resource.

### The prognostic and clinical information of PDCD2 in pan-cancer

We used the GEPIA database (http://gepia.cancer-pku.cn/)^18^ and the PrognoScan database (http://dna00.bio.kyutech.ac.jp/PrognoScan/index.html)^19^ to examine the prognostic values of PDCD2 in solid cancers. A cut-off value of 50% was used to divide cohorts with high and low expression.

### The CCDC50 gene mutation in pan-cancer

Through the cBioPortal (https://www.cbioportal.org/),^20^ we analyzed the mutation information in human cancer (excluded hematopoietic malignancies) for the PDCD2 gene.

### Curve analysis of receiver operating characteristics (ROC)

To assess the diagnostic value of screening signature genes, we utilized the pROC function from the R package to generate receiver operating characteristic (ROC) curves and calculate the area under the curve (AUC).

### Gene set enrichment analysis

Gene set enrichment analysis (GSEA) software (version 3.0) was obtained from GSEA^21^ and samples were divided into high expression (≥50%) and low expression (<50%) groups according to the PDCD2 expression level. The c2.cp.kegg.v7.4.symbols.gmt subcollection was downloaded from the Molecular Signatures Database^22^ (http://www.gsea-msigdb.org/gsea/downloads.jsp). The minimum gene set is 5, the maximum gene set is 5000, and a thousand resamples were used to evaluate the related pathways and molecular mechanisms; a false discovery rate (FDR) < 0.05 and a *P* value < 0.05 were considered statistically significant.

### Analysis of the protein and gene interaction networks

GeneMANIA^23^ (http://www.genemania.org) and STRING^24^ (https://string-db.org/) were utilized to build a network of interactions between genes and proteins associated with PDCD2.

### Cell lines and cell culture

U251 and U87 glioma cell lines were bought from the Chinese Academy of Sciences (Shanghai, China) with STR document, and cultured in DMEM media (Lonza, CC3170) supplemented with 10% fetal bovine serum (FBS) and 1% penicillin/streptomycin.

### Small interfering RNA (siRNA)

The siRNAs for PDCD2 (si-PDCD2#1: 5′-UGGGUAGUUGAUUCCUAAAAACUCGCA-3′; si-PDCD2#2: 5′-UAUAAUCUCUGAGUAAUCUUCCUUUUC-3′) were synthesized by ORIGENE. As a negative control, scrambled siRNA was synthesized. Lipofectamine 3000 (Invitrogen) was used for transfection.

### Quantitative real-time PCR

The qRT-PCR assay was performed as previously described.^25^ The primer sequences were as follows: PDCD2-F: 5′-ACT CAT ATG AGC CAC CTT CTG AG-3′; PDCD2-R: 5′-GTC TGA TGC TCC TTG CTG CAG-3′; β-actin-F: 5′-TCC TGT GGC ATC CAC GAA ACT-3′; β-actin-R: 5′-GAA GCA TTT GCG GTG GAC GAT-3′. Expression quantification was obtained with the 2^−ΔΔCT^ method.

### Western blotting

Western blotting was performed as previously described.^26^ The primary antibodies used included PDCD2 (Proteintech, 10725-1-AP) and β-actin (sc-47778, Santa Cruz). Using a chemiluminescence kit (Amersham Pharmacia Biotech, Piscataway, NJ), protein bands were detached. All the experiments were repeated thrice.

### Cell proliferation, colony formation assay, and transwell assay

At 0, 24, 48, 72, and 96 h after cell seeding, absorbance (at 450 nm) in each well was determined with CCK8 kit (C0037, Beyotime).^27^ For colony formation assays, seeded cells in 6-well plates (800 cells/well) were then cultured for 10 days. Transwell assays were performed as previously described.^26^^,^^28^ All the experiments were repeated thrice.

### Xenograft model studies

Male BALB/*c* nude mice, aged 4 weeks, were obtained from GemPharmatech Technology (Jiangsu, China). Each mouse's left flank was injected with U87 cells at a concentration of 4 × 10^6^ cells/mouse. Nine mice were injected with si-NC or siRNA (10 nmol in 0.1 mL of saline buffer per tumor nodule) every 2 days for a total of nine injections. After 10 days, the xenograft models were divided into three groups (*n* = 3) once the tumors reached a size of approximately 6 mm × 5 mm. On Day 34, the tumor-bearing mice were euthanized by cervical dislocation after being anesthetized with isoflurane. The xenograft tumors were then dissected, weighed, and photographed.

### Immunohistochemistry

The technique of immunohistochemistry (IHC) was carried out following the methods described earlier.^28^^,^^29^ The xenograft sections of nude mice were treated with primary antibodies against PDCD2 (Proteintech, 10725-1-AP) and Ki67 (sc-23900, Santa Cruz) for overnight incubation at 4 °C. Subsequently, they were exposed to a secondary antibody.

### Statistical analysis

*T*-tests were used to estimate PDCD2 expression in pan-cancer. Correlations between clinicopathological characteristics and PDCD2 expression were assessed using *Chi*-square tests, Fisher's exact tests, Kruskal–Wallis tests, and Wilcoxon's signed rank tests. Cox logistic regression models with univariate and multivariate analyses were used to uncover clinical factors that affected survival and PDCD2 expression. The survival time of patients with high or low PDCD2 expression was analyzed by Kaplan-Meier analysis. The symbols ns, ^∗^, ^∗∗^, and ^∗∗∗^ indicate *P* > 0.05, *P* < 0.05, *P* < 0.01, and *P* < 0.001, respectively.

## Results

### Analysis of PDCD2 expressed in solid cancers

**Figure 1:**
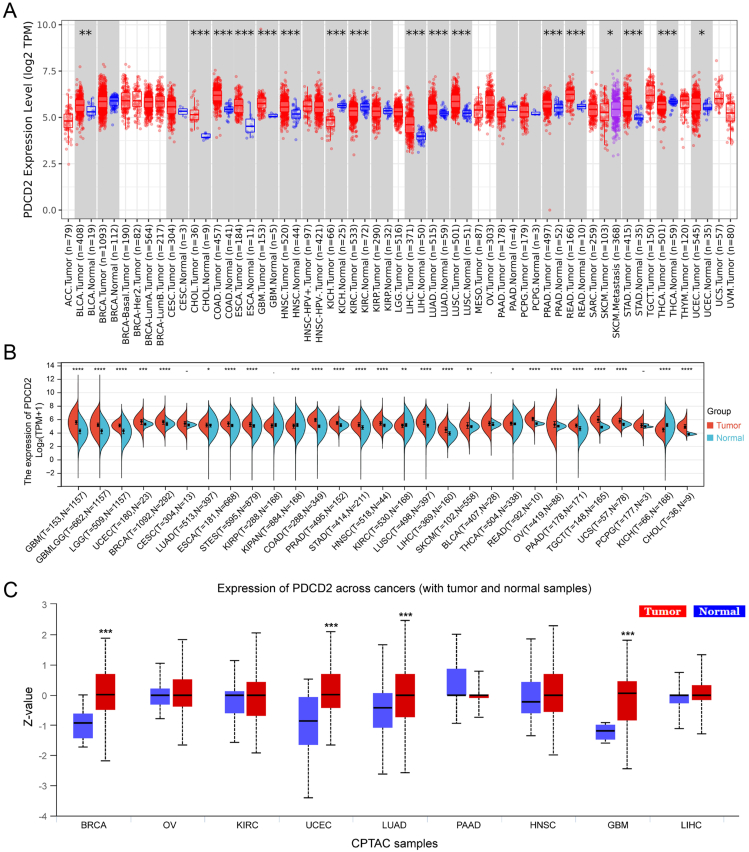
PDCD2 expression in pan-cancer. **(A)** Examining the TIMER database uncovers PDCD2 expression in pan-cancers. **(B)** Pan-cancer analysis of PDCD2 expression in TCGA/GTEx. **(C)** Data from the UALCAN database on PDCD2 protein in pan-cancer analysis. ns, *P* > 0.05; ^∗^*P* < 0.05, ^∗∗^*P* < 0.01, ^∗∗∗^*P* < 0.001.

First, analysis of the TIMER database was carried out to examine the PDCD2 expression level in human solid cancers. We found that PDCD2 expression was increased in bladder urothelial carcinoma (BLCA), stomach adenocarcinoma (STAD), cholangiocarcinoma (CHOL), colon adenocarcinoma (COAD), glioblastoma multiforme (GBM), rectum adenocarcinoma (READ), liver hepatocellular carcinoma (LIHC), lung adenocarcinoma (LUAD), esophageal carcinoma (ESCA), lung squamous cell carcinoma (LUSC), prostate adenocarcinoma (PRAD), breast invasive carcinoma (BRCA), and uterine corpus endometrial carcinoma (UCEC) tissues compared with adjacent normal tissues. In addition, low expression of PDCD2 in cancer was observed in kidney clear cell carcinoma (KIRC), kidney chromophobe (KICH), and thyroid carcinoma (THCA) (Fig. 1A).

Next, we combined the TCGA and GTEx database, and the results confirmed that PDCD2 expression was significantly higher in GBM, GBMLGG, brain lower grade glioma (LGG), UCEC, BRCA, LUAD, stomach and esophageal carcinoma (STES), COAD, PRAD, STAD, head and neck squamous cell carcinoma (HNSC), LUSC, LIHC, skin cutaneous melanoma (SKCM), THCA, rectum adenocarcinoma (READ), esophageal carcinoma (ESCA), ovarian serous cystadenocarcinoma (OV), pancreatic adenocarcinoma (PAAD), testicular germ cell tumors (TGCT), uterine carcinosarcoma (UCS), and CHOL than in paired adjacent normal tissues (Fig. 1B). In order to investigate the level of PDCD2 protein in human solid cancers, we used UALCAN database analysis and proved that PDCD2 was highly expressed in BRCA, UCEC, LUAD, and GBM (Fig. 1C).

### Prognostic values of PDCD2 in solid cancers

**Figure 2:**
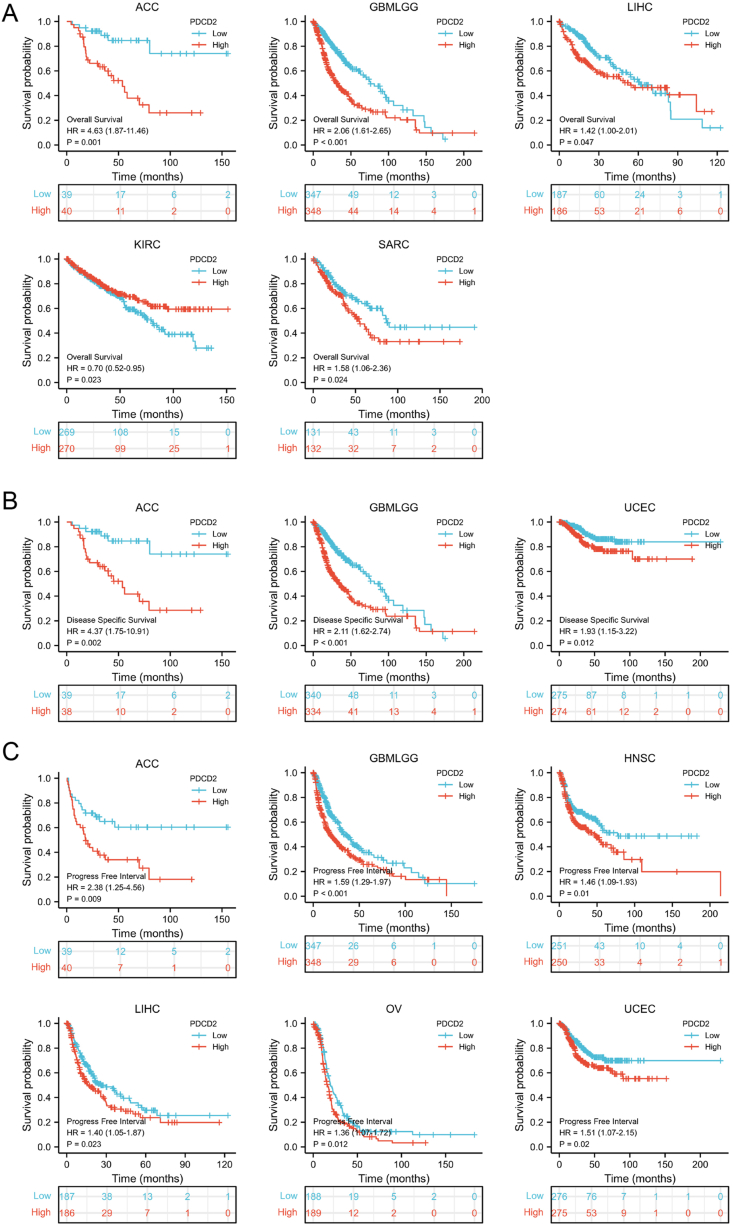
The PDCD2 prognostic values in pan-cancer. **(A)** The impact of PDCD2 expression on the overall survival rate of patients with ACC, GBMLGG, LIHC, KIRC, or SARC. **(B)** The disease-specific survival for PDCD2 in ACC, GBMLGG, and UCEC. **(C)** The progress-free survival for PDCD2 in ACC, GBMLGG, HNSC, LIHC, OV, and UCEC.

Since PDCD2 expression was differently expressed in many types of cancer, the ability of prognostic values of PDCD2 in human cancer was explored. A higher level of PDCD2 expression was associated with lower overall survival (OS) for adrenocortical carcinoma (ACC), GBMLGG, LIHC, and Sarcoma (SARC) (Fig. 2A), poor disease-specific survival (DSS) in ACC, GBMLGG, and UCEC (Fig. 2B), and poor progression-free interval (PFI) in ACC, GBMLGG, HNSC, LIHC, OV, and UCEC (Fig. 2C).

### PDCD2 could act as a potential biomarker in solid cancers

**Figure 3:**
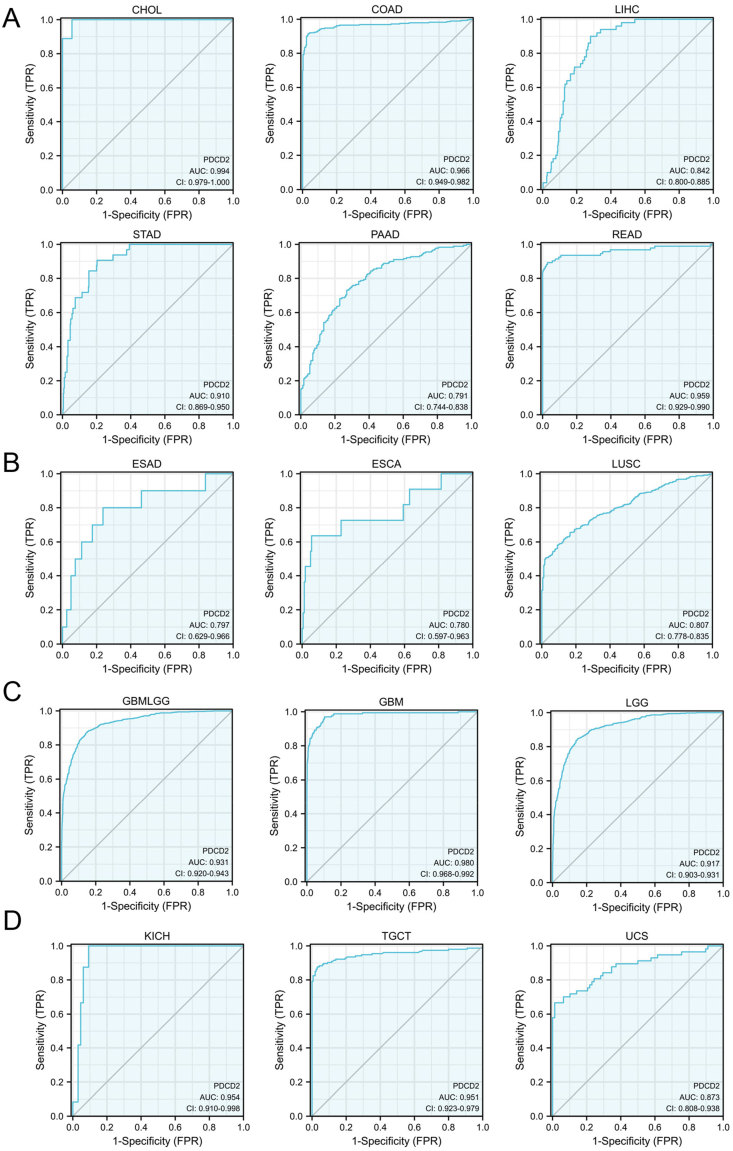
ROC curve analysis of PDCD2 in pan-cancer. ROC curve analysis of PDCD2 in CHOL, COAD, LIHC, STAD, PAAD, and READ **(A)**, ESAD, ESCA, and LUSC **(B)**, GBMLGG, GBM, and LGG **(C)**, KICH, TGCT, and UCS **(D)**. TPR, true positive rate; FPR, false positive rate.

To investigate whether PDCD2 could act as a biomarker in solid cancers, the ROC curve analysis was conducted, and the results indicated that PDCD2 could be used as a high sensitivity and specificity biomarker (AUC > 0.75) for diagnosing digestive system tumors such as CHOL, COAD, STAD, PAAD, and READ (Fig. 3A), chest tumors including esophageal adenocarcinoma (ESAD), ESCA, and LUSC (Fig. 3B), nervous system tumor GBMLGG including GBM and LGG (Fig. 3C), and genitourinary system tumors including KICH, TGCT, and UCS (Fig. 3D).

### Gene mutation landscape of PDCD2 in solid cancers

**Figure 4:**
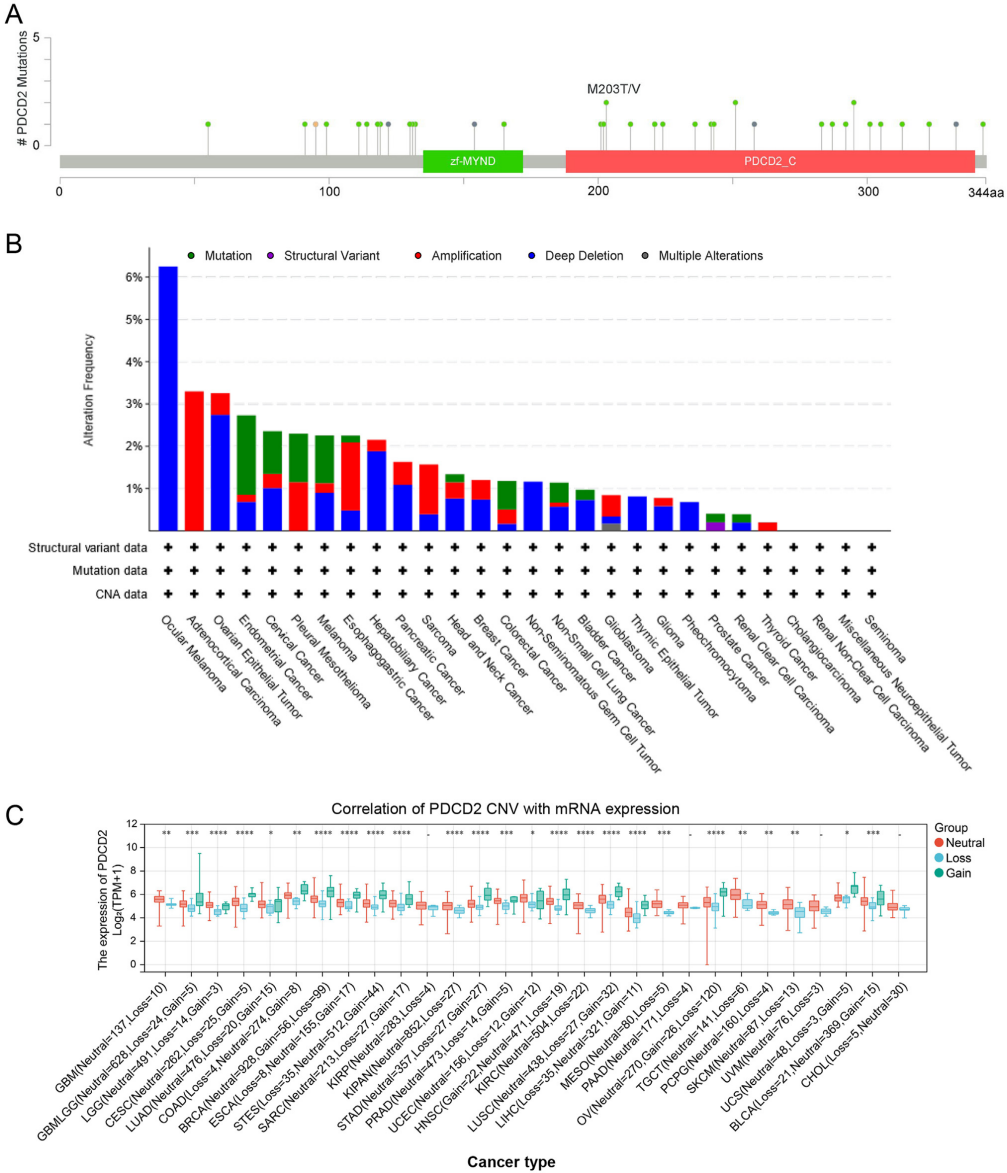
Mutational analysis of PDCD2. **(A)** Hot spots of mutation of PDCD2. **(B)** A summary of PDCD2 mutation types and their distribution among different cancers. **(C)** Correlation of PDCD2 CNV with mRNA expression in pan-cancer. ns, *P* > 0.05; ^∗^*P* < 0.05, ^∗∗^*P* < 0.01, ^∗∗∗^*P* < 0.001.

Data on PDCD2 mutations was downloaded from the cBioPortal. To analyze the PDCD2 mutation landscape in human cancer, 33 missense sites, 4 truncation sites, and 1 fusion situated between amino acids 0 and 344 were identified in PDCD2 (Fig. 4A). In addition, ocular melanoma, ACC, and the ovarian epithelial tumor had a higher mutation frequency than other cancers (Fig. 4B). Furthermore, we found that copy number variations (CNV) of PDCD2 regulate gene expression through different means (increasing or decreasing) in diverse cancer types (Fig. 4C). According to these results, PDCD2 has different mutation forms in different tumor types.

### PDCD2 as an independent prognostic factor for GBMLGG

**Figure 5:**
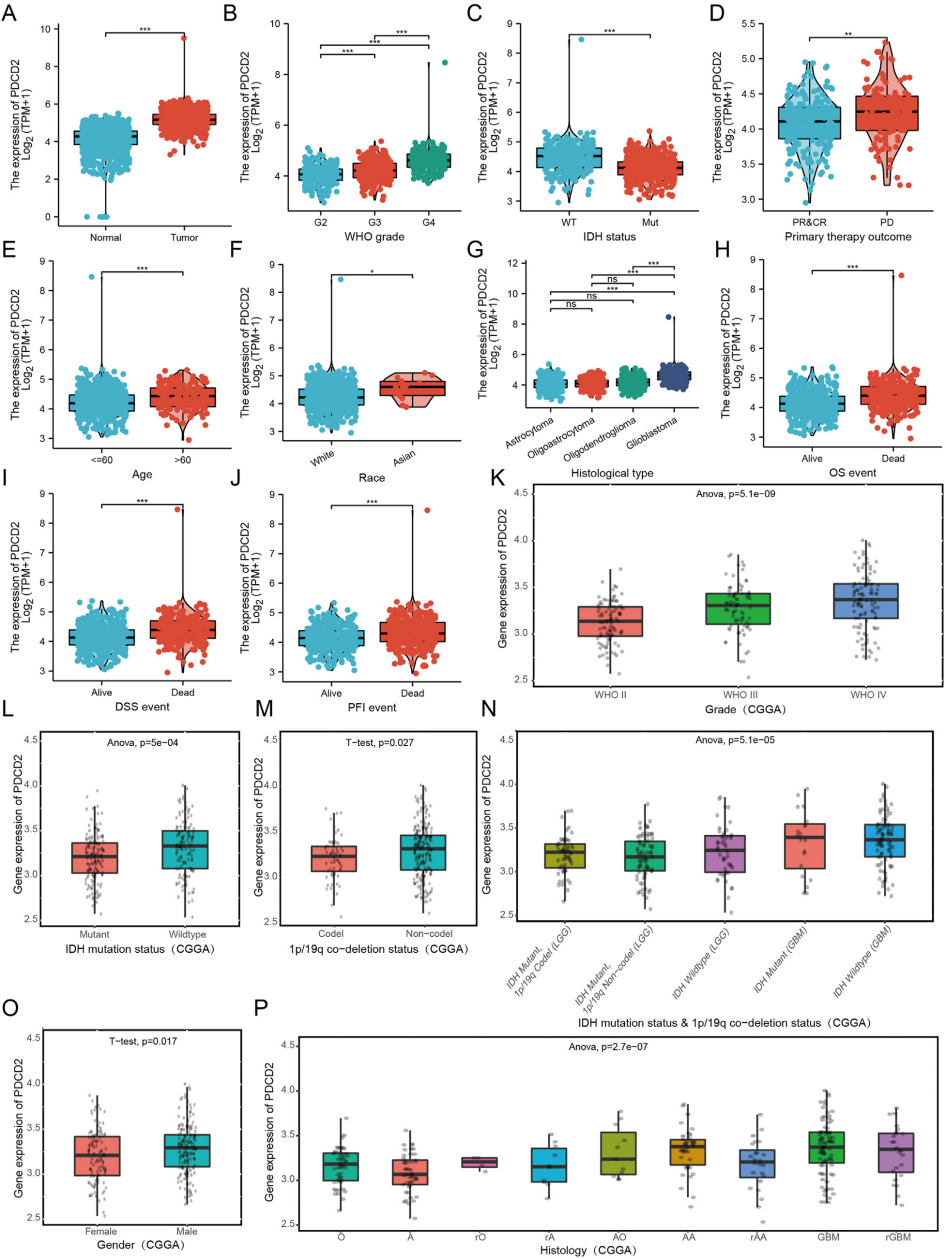
PDCD2 is correlated with clinical characteristics in GBMLGG. The PDCD2 was up-regulated in GBMLGG **(A)**, and associated with WHO grade, IDH status, primary therapy outcome, age, race, histological type, OS event, DSS event, and PFI event **(B–J)** (data from TCGA). PDCD2 is correlated with clinical characteristics in glioma, including the WHO grade, IDH mutation status, 1p/19q co-deletion status, IDH mutation status & 1p/19q co-deletion status, gender, and histology **(K–P)** (data from CGGA). G2, grade II; G3, grade III; G4, grade IV; PR, partial response; CR, complete response; PD, progressive disease; OS, overall survival; DSS, disease-specific survival; PFS, progression-free survival; WT, wild type; Mut, mutant. ^∗^*P* < 0.05, ^∗∗^*P* < 0.01, ^∗∗∗^*P* < 0.001.

**Figure S1:**
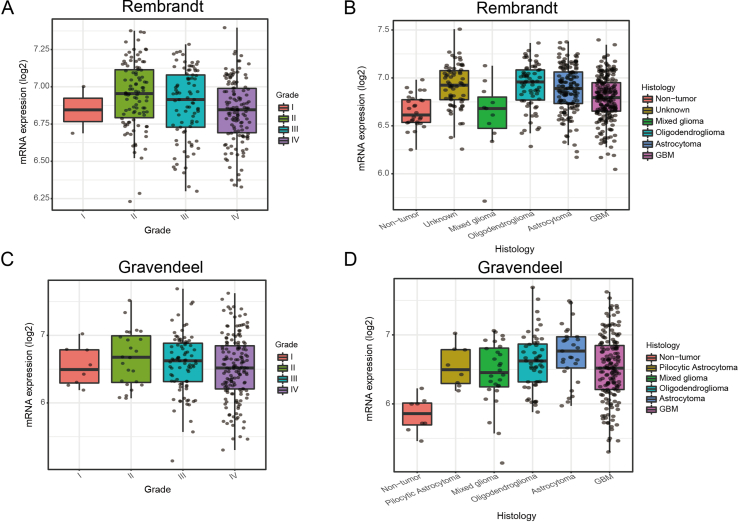
Examining the relationship between GBMLGG clinical characteristics and PDCD2 expression levels through correlation analysis. **(A–D)** Correlation analysis between PDCD2 expression levels and GBMLGG grade/histology in the Rembrandt database (A, B) and the Gravendeel database (C, D).

It appears that PDCD2 plays an important biological role in GBMLGG. Next, we examined the relationship between PDCD2 expression and GBMLGG clinical features. Data from TCGA showed that high PDCD2 expression was strongly associated with WHO grade, IDH status, primary therapy outcome, age, race, histological type, OS event, DSS event, and PFI event (Fig. 5A–G). Similarly, CGGA database^15^ indicated that high PDCD2 expression was significantly associated with 1p/19q co-deletion status, grade, IDH mutation status, gender, and IDH mutation status & 1p/19q co-deletion status (Fig. 5K–P). Using the Rembrandt database^30^ and the Gravendeel database,^31^ an association between PDCD2 expression and GBMLGG grade and histology also was found (Fig. S1).

**Figure 6:**
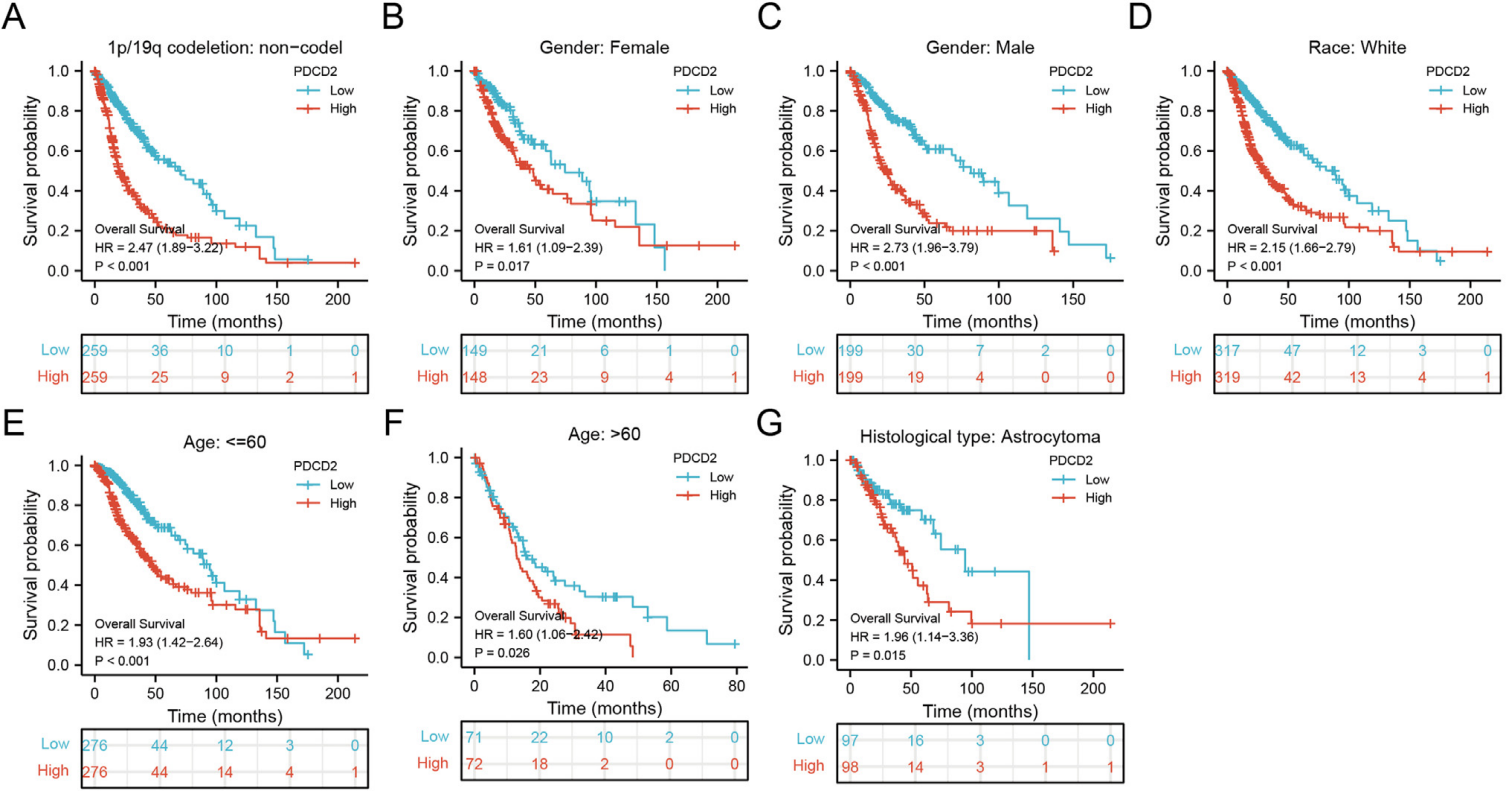
PDCD2 is correlated with prognosis in GBMLGG. The correlation between PDCD2 and OS in 1p/19q co-deletion status **(A)**, gender (both female and male) **(B, C)**, race (white) **(D)**, age **(E, F)**, and histological type **(G)** of GBMLGG.

**Figure S2:**
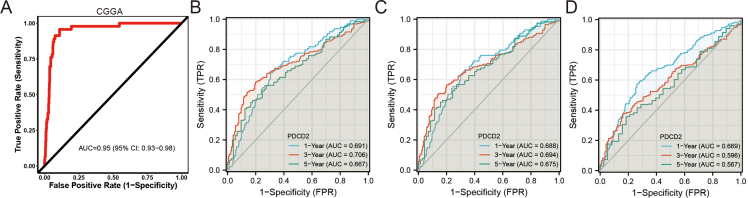
ROC curve analysis of PDCD2 in GBMLGG. **(A)** ROC curve analysis of PDCD2 in GBMLGG was examined by the CGGA database. The OS **(B)**, DSS **(C)**, and PFI **(D)** of PDCD2 in GBMLGG were examined by the TCGA database.

We continued to explore the prognostic analysis of GBMLGG patients and PDCD2 expression in TCGA. In patients with many clinical features, including 1p/19q co-deletion status, gender (both female and male), race, age, and histological type, those with high PDCD2 expression have a poor prognosis (Fig. 6A–G). To investigate whether PDCD2 could act as a biomarker in GBMLGG, the ROC curve analysis was performed, and the results indicated that PDCD2 could be used as a high-sensitivity and specificity biomarker to diagnose GBMLGG (Fig. S2A–D). Univariate and multivariate Cox regression analysis revealed that WHO grade, IDH status, primary therapy outcome, age, histological type, and PDCD2 expression were significantly associated with OS (Table 1).

**Table 1:** Univariate and multivariate Cox regression analyses of clinical characteristics associated with OS of glioma.

Characteristics	Total (*N*)	Univariate analysis		Multivariate analysis	
		Hazard ratio (95% CI)	*P* Value	Hazard ratio (95% CI)	*P* Value
WHO grade	634				
G2	223	Reference			
G3	243	2.999 (2.007–4.480)	**<0.001**	2.638 (0.988–7.042)	0.053
G4	168	18.615 (12.460–27.812)	**<0.001**	5.795 (1.142–29.396)	**0.034**
IDH status	685				
WT	246	Reference			
Mut	439	0.117 (0.090–0.152)	**<0.001**	0.322 (0.144–0.721)	**0.006**
Primary therapy outcome	461				
PD	112	Reference			
SD	147	0.440 (0.294–0.658)	**<0.001**	0.430 (0.180–1.025)	0.057
PR	64	0.170 (0.074–0.391)	**<0.001**	0.272 (0.078–0.953)	**0.042**
CR	138	0.133 (0.064–0.278)	**<0.001**	0.210 (0.069–0.634)	**0.006**
Age	695				
≤60	552	Reference			
>60	143	4.668 (3.598–6.056)	**<0.001**	4.252 (1.924–9.397)	**<0.001**
Histological type	363				
Astrocytoma	195	Reference			
Glioblastoma	168	6.160 (4.446–8.535)	**<0.001**		
PDCD2	695	2.183 (1.840–2.590)	**<0.001**	1.149 (0.615–2.147)	0.664

Bold value represents significant values.

**Figure 7:**
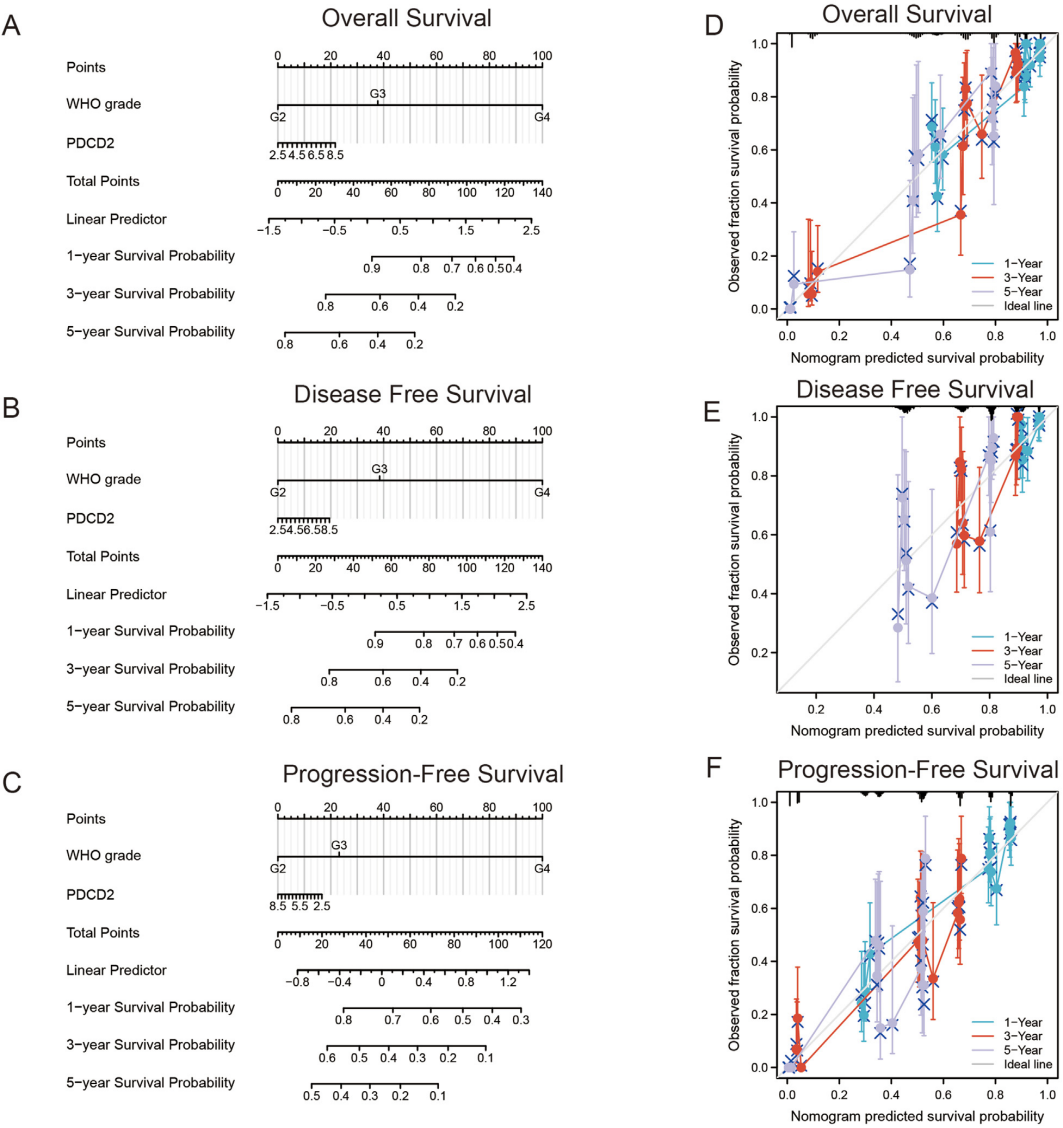
The ability of PDCD2 to predict the GBMLGG patients' prognosis. Construction and evaluation of nomogram used for predicting OS **(A)**, DFS **(B)**, and PFS **(C)** of GBMLGG patients. The calibration curve and Hosmer–Lemeshow test of nomograms in the TCGA-GBMLGG cohort for OS **(D)**, DFS **(E)**, and PFS **(F)**.

The TCGA-GBMLGG cohort was also used to construct a nomogram to predict OS, disease-free survival (DFS), and progression-free survival (PFS). In the nomogram, pathological stage and PDCD2 expression acted as prognostic factors (Fig. 7A–C). The calibration curves indicated that the nomogram could reliably predict 1-, 3-, and 5-year OS, DFS, and PFS in GBMLGG (Fig. 7D–F). In summary, the nomogram has a good ability to predict the GBMLGG patients' prognosis.

### Interaction network of PDCD2 in GBMLGG

**Figure 8:**
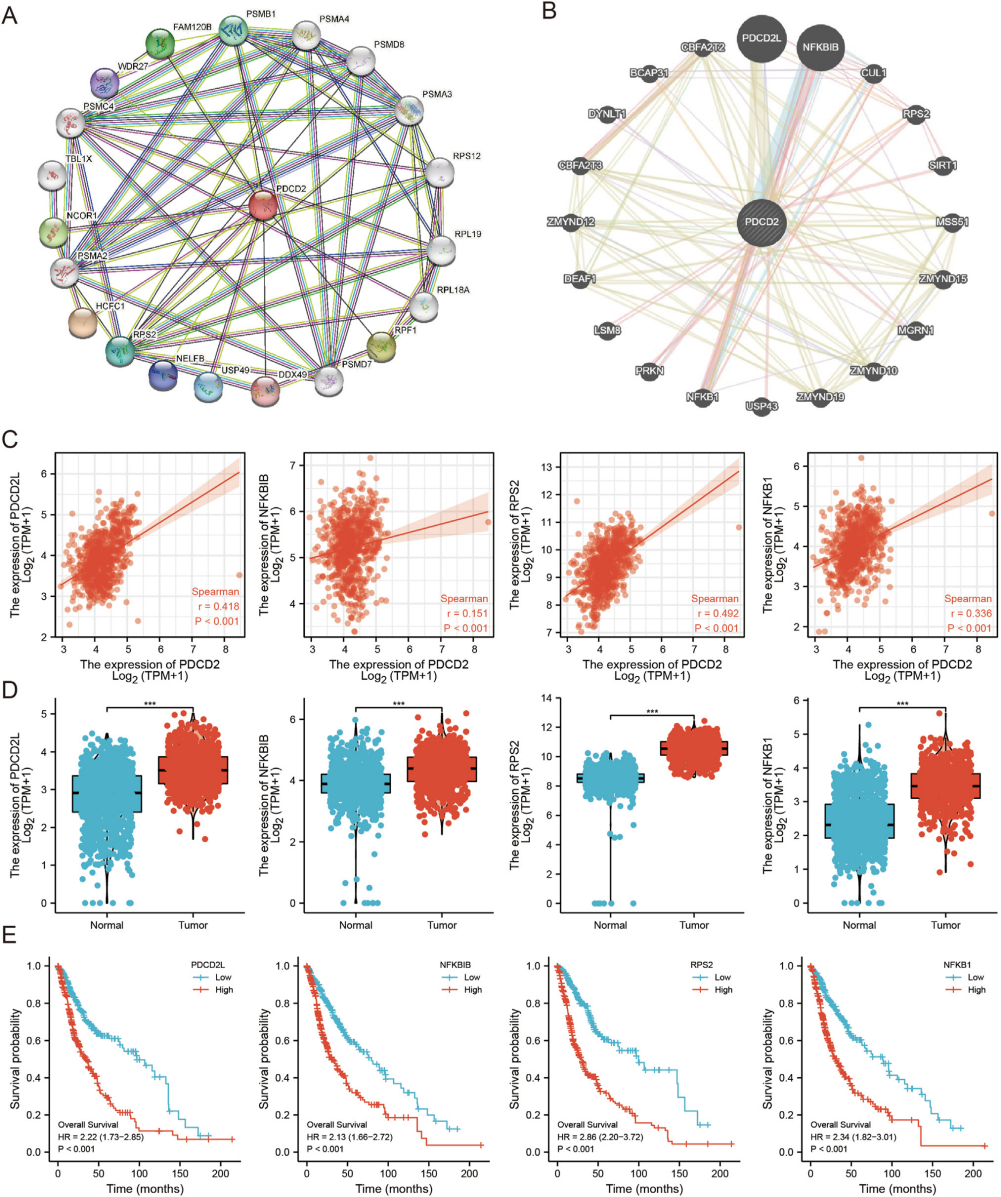
Interaction network of PDCD2. The network of PDCD2 at the protein **(A)** and gene **(B)** levels in pan-cancer. Correlation between PDCD2 expression and the expression of PDCD2L, NFKBIB, PRS2, and NFKB1 in GBMLGG **(C)**. **(D, E)** Expression and prognosis of PDCD2L, NFKBIB, PRS2, and NFKB1 in GBMLGG.

**Figure 9:**
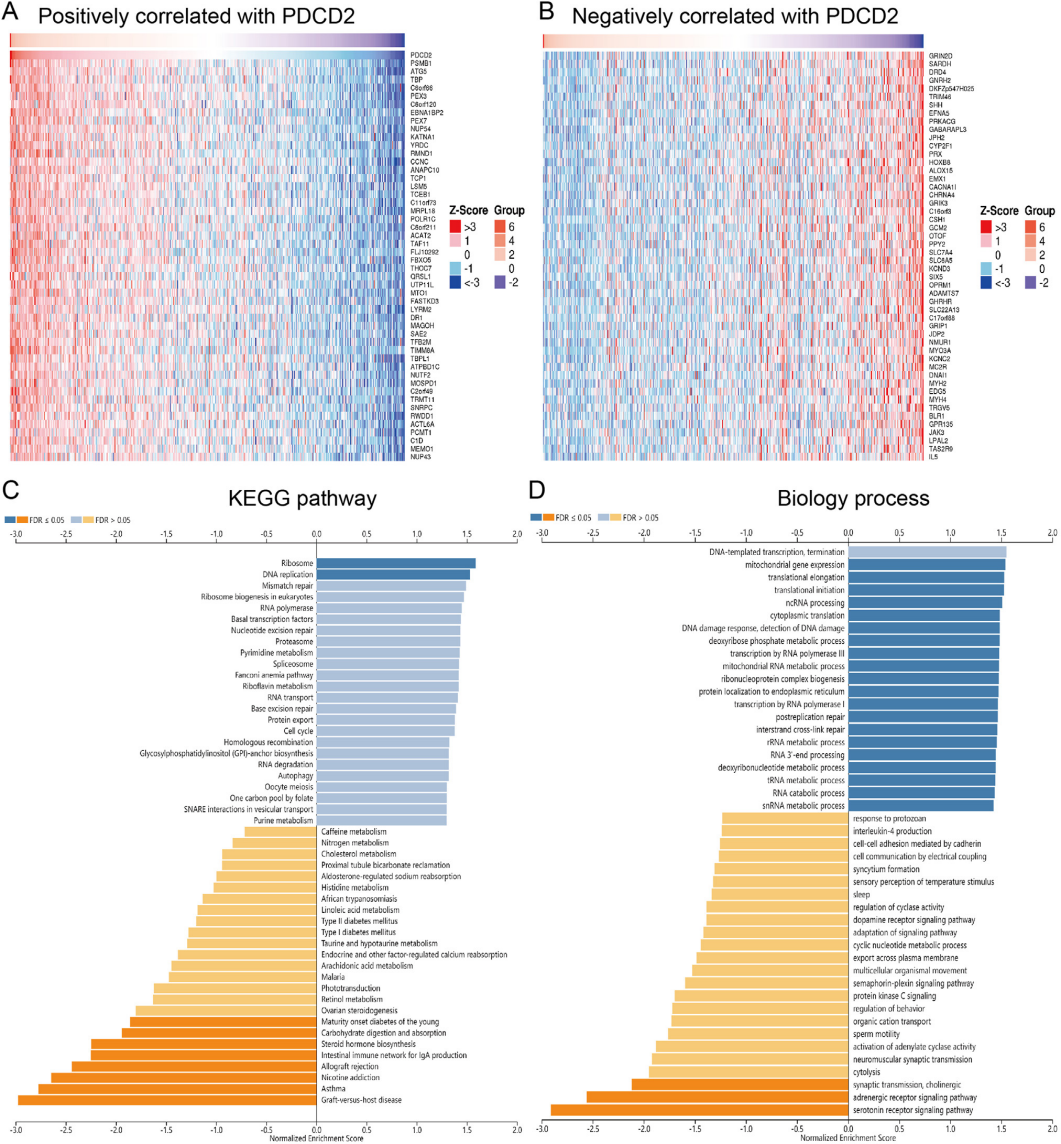
KEGG and GO enrichment analysis for PDCD2. The top 50 genes positively **(A)** and negatively **(B)** correlated with PDCD2 are shown in the heat map. **(C, D)** KEGG pathway (C) and biology process analysis (D) of PDCD2.

The network of PDCD2 at the protein and gene levels in pan-cancer was shown in Figure 8A and 8B. After examined the association between these genes and PDCD2 in GBMLGG, we discovered a noteworthy positive correlation between PDCD2 and the expression of PDCD2L, NFKBIB, PRS2, and NFKB1 as depicted in Figure 9C. Additional analysis revealed that GBMLGG exhibited significant up-regulation of the four genes, which was associated with a poorer prognosis among patients (Fig. 8D, E).

### Function analysis of PDCD2 in GBMLGG

Using Linked-omics, we performed a correlation analysis of PDCD2 in GBMLGG to further determine its possible function. A heat map was constructed to illustrate the genes whose expression was more positively and negatively correlated with that of PDCD2 (Fig. 9A, B). KEGG pathway analysis showed the enrichment in the ribosome, DNA replication, asthma, nicotine addiction, allograft rejection, the intestinal immune network for IgA production, carbohydrate digestion and absorption, and maturity-onset diabetes of the young (Fig. 9C). GO annotation revealed these genes' participation in mitochondrial elongation, translational initiation, ncRNA processing, cytoplasmic translation, DNA damage response, detection of DNA damage, deoxyribose phosphate metabolic process, transcription by RNA polymerase III, mitochondrial RNA metabolic process, ribonucleoprotein complex biogenesis, protein localization to the endoplasmic reticulum, transcription by RNA polymerase I, post replication repair, interstrand cross-link repair, rRNA metabolic process, RNA 3′-end processing, deoxyribonucleotide metabolic process, RNA catabolic process, snRNA metabolic process, serotonin receptor signaling pathway, adrenergic receptor signaling pathway, and synaptic transmission (Fig. 9D).

### GSEA identification of PDCD2-associated signaling pathways in GBMLGG

**Figure 10:**
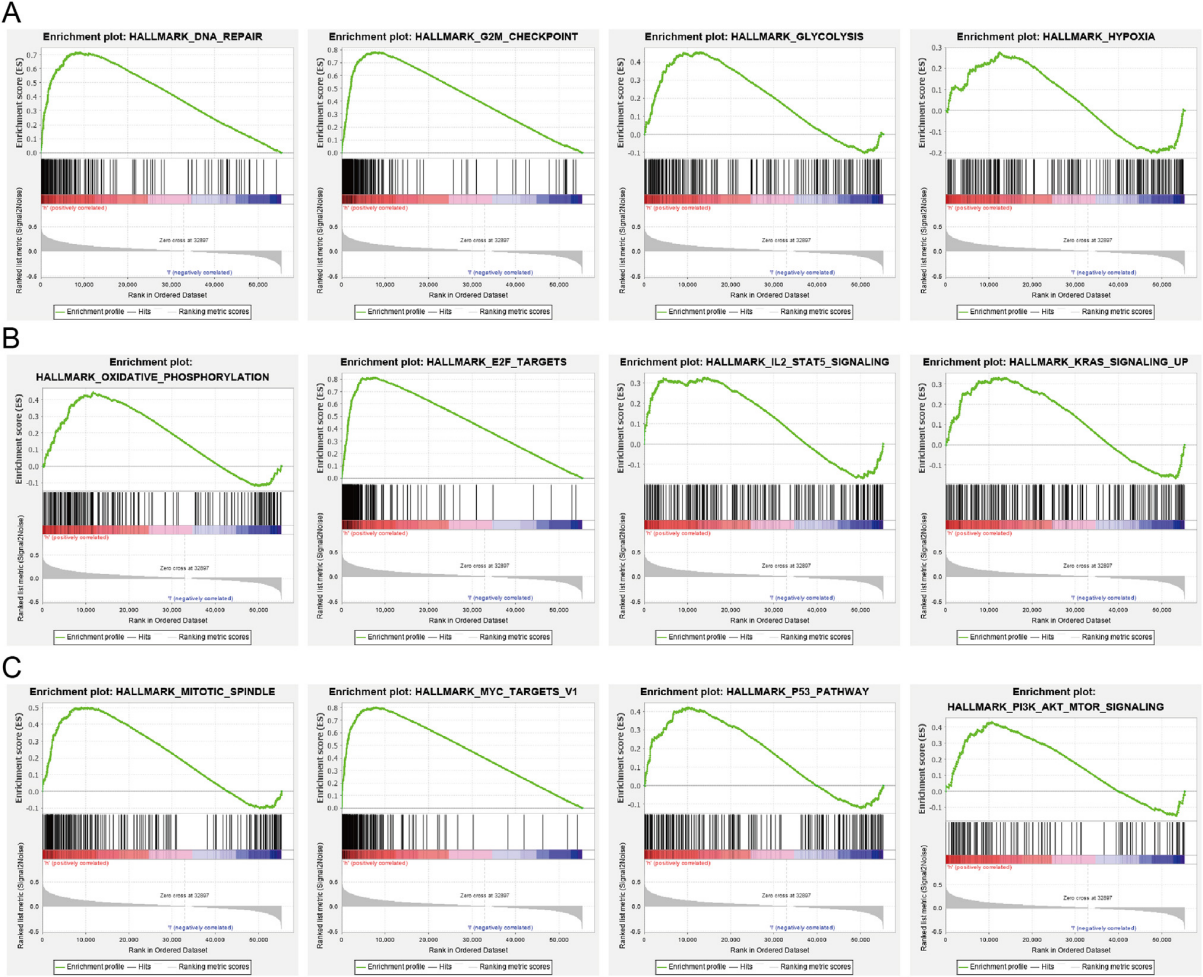
GSEA enrichment PDCD2-related signaling pathway in GBMLGG.

**Figure 11:**
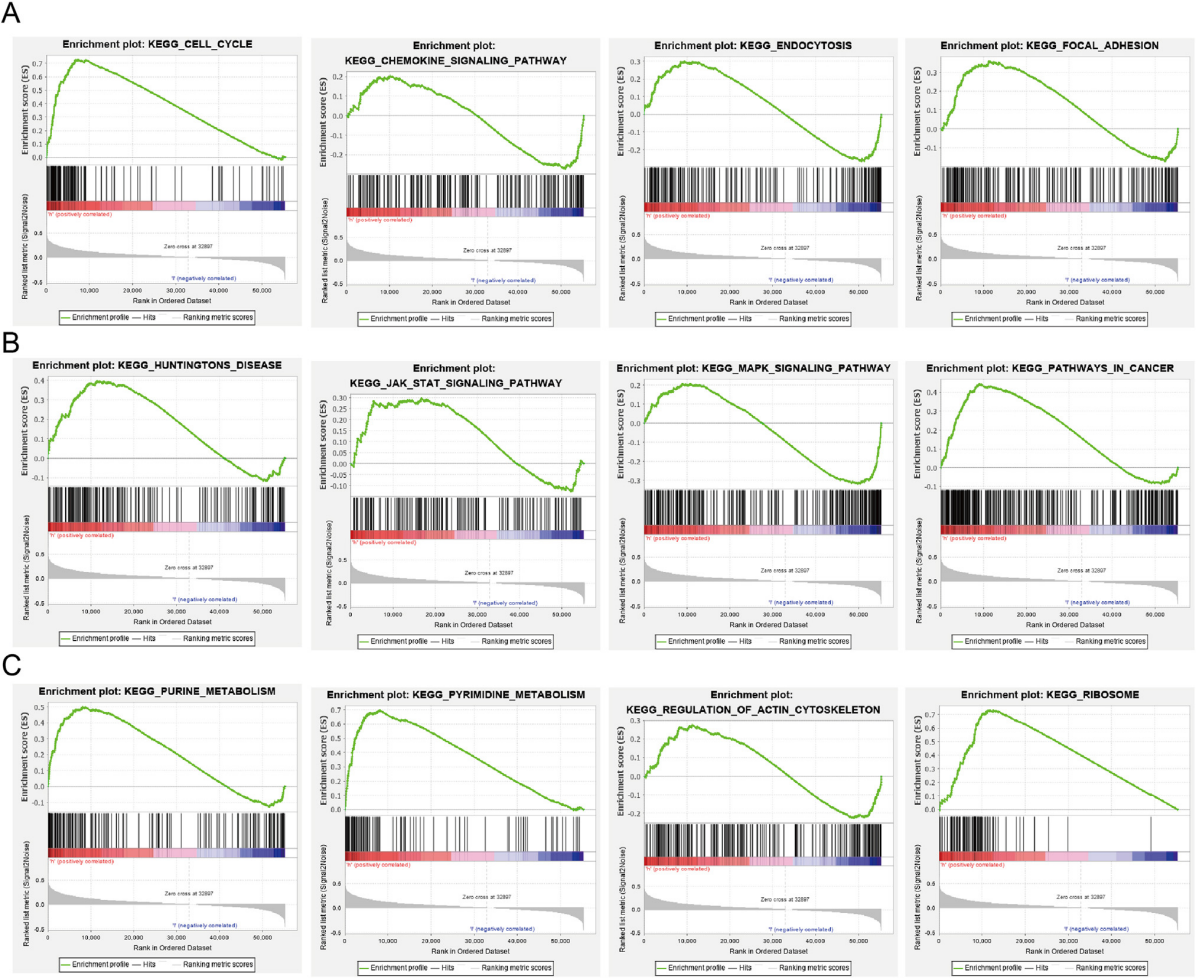
KEGG enrichment PDCD2-related signaling pathway in GBMLGG.

PDCD2 participates mainly in the biological processes associated with GBMLGG, according to the GSEA-enriched analysis we performed. Hallmark characteristic enriched results indicated that PDCD2 was enriched primarily in the regulation of DNA repair, G2M checkpoint, P53 pathway, glycolysis, hypoxia, oxidative phosphorylation, E2F targets, IL2 stat5 signaling, MYC targets, KRAS signaling, mitotic spindle, and PI3K/AKT/mTOR signaling (Fig. 10). In addition, KEGG enriched results showed that PDCD2 was mainly involved in cell cycle, pyrimidine metabolism, purine metabolism, endocytosis, focal adhesion, Huntington's disease, JAK/STAT signaling pathway, MAPK signaling pathway, chemokine signaling pathway, pathways in cancer, regulation of actin cytoskeleton, and ribosome (Fig. 11A–C).

### PDCD2 promotes GBMLGG cell migration, invasion, and xenograft tumor growth

**Figure 12:**
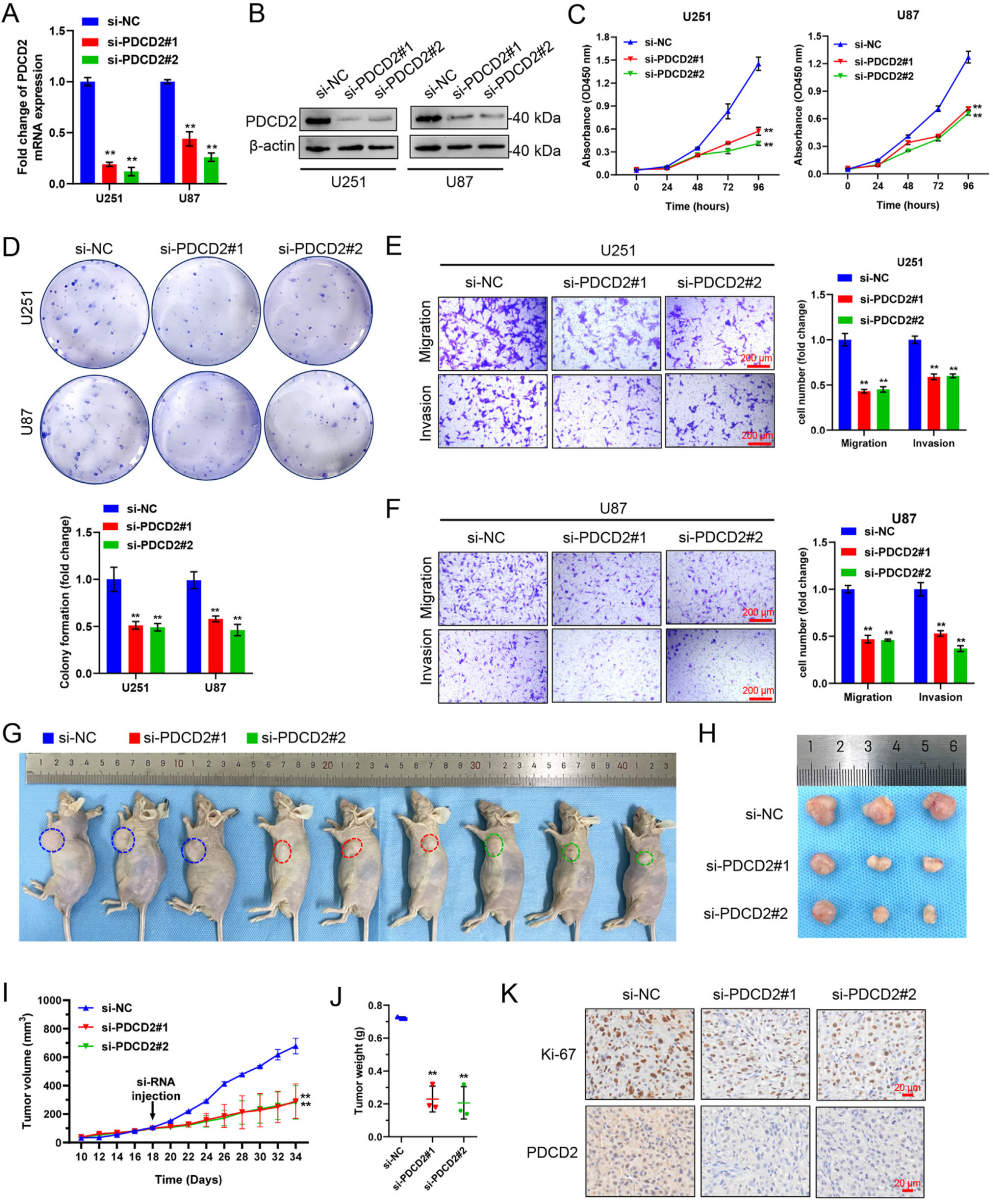
PDCD2 promotes GBMLGG progression. **(A, B)** To determine the levels of PDCD2 expression in U251 and U87 cells transfected with siRNA targeting PDCD2, qPCR and Western blotting were employed. **(C)** Cell growth ability was assessed using CCK8 assays at the specified time intervals in U251 and U87 cells transfected with PDCD2 siRNAs. **(D)** PDCD2 knockdown inhibited colony formation of both U251 and U87 cells. **(E, F)** The migration and invasion of U251 and U87 cells were found to be inhibited by PDCD2 knockdown, as demonstrated by the transwell migration and invasion assays (scale bars = 200 μm). U87 cells were injected subcutaneously into nude mice to establish a xenograft model. Following the development of the tumor, intertumoral injection of si-PDCD2 or NC was performed (*n* = 3/group). **(G**–**I)** Representative pictures and tumor growth chart at 16 days post-injection of si-PDCD2 or NC in GBMLGG xenograft model. **(J)** The weight of the xenograft tumor was measured and graphed 16 days after the injection. **(K)** IHC staining was used to examine the alterations in the levels of PDCD2 and Ki-67 in xenograft tumors. Representative images are shown (scale bars = 20 μm). The data were presented as mean ± standard deviation of three independent experiments. ^∗^*P* < 0.05, ^∗∗^*P* < 0.01, ^∗∗∗^*P* < 0.001.

First, we selected GBMLGG cell lines U251 and U87 with a high malignant degree. The next step was to design and synthesize two siRNAs that specifically targeted PDCD2. U251 and U87 cell lines were transfected with siRNA, and qPCR and Western blotting were conducted to assess knockdown efficiency. Both siRNAs were significantly effective in reducing PDCD2 expression (Fig. 12A, B). Using the CCK-8 assay, we also found that knocking down PDCD2 significantly inhibited the viability of GBMLGG cells (Fig. 12C). Colony formation was significantly inhibited in colonies formed by U251 and U87 cells when PDCD2 was knocked down (Fig. 12D). We also examined the impact of PDCD2 on the migration and invasion of GBMLGG cells using transwell experiments. The results indicated that the suppression of PDCD2 greatly decreased migration and invasion (Fig. 12E, F). Further, we examined the impact of PDCD2 on GBMLGG in an *in vivo* setting by utilizing a xenograft tumor model. Tumors were injected with NC or PDCD2 siRNA every other day, starting from Day 18, and the mice were euthanized on Day 34. The tumor growth curve indicated that the growth rate of xenograft tumors created by U87 cells with reduced PDCD2 levels was noticeably decreased in comparison to the NC (Fig. 12G–I). The xenograft tumors that received si-PDCD2 had a lower weight compared with those in the NC group (Fig. 12J). In si-PDCD2 injected xenograft tumors, IHC staining indicated a reduction in the expression of Ki67 and PDCD2 compared with the NC group (Fig. 12K). These findings indicate that PDCD2 might be responsible for GBMLGG's oncogenic effects.

## Discussion

Comparing heterogeneity between various tumors through pan-cancer analysis is essential for the discovery of new cancer biomarkers and therapeutic targets.^32^ The involvement of PDCD2 in the development of lymphomas in human B and T cells has been shown.^10^ PDCD2 can also predict the occurrence of leukemia.^8^ At present, there have been no investigations conducted to determine if PDCD2 is linked to overall cancer prognosis.

During this study, it was observed that PDCD2 expression levels were notably elevated in various forms of malignancies. Furthermore, a strong association was identified between PDCD2 expression and unfavorable OS rates in cases of ACC, GBMLGG, LIHC, and SARC. Similarly, a negative correlation was found between PDCD2 expression and DSS rates in ACC, GBMLGG, and UCEC, as well as poor PFI rates in ACC, GBMLGG, HNSC, LIHC, OV, and UCEC. ROC curve analysis indicates that PDCD2 could serve as a highly specific and sensitive biomarker for the diagnosis of different types of cancers.

In addition, our team verified the correlation between PDCD2 and genetic mutation. The highest frequency of PDCD2 alterations (>6%) was observed in ocular melanoma. Additionally, we verified that the rates of modification were 3.3%, 3.25%, and 2.73% in ocular melanoma, ACC, and ovarian epithelial tumors, respectively.

Glioma is a common malignant tumor of the central nervous system. This tumor type has the characteristics of rapid growth and poor prognosis. In addition, the clinical treatment effect is not satisfactory.^1^^,^^33^ In the TCGA GBMLGG cohort, decreased levels of PDCD2 expression and pathological stage were significantly associated with better OS, DSS, and PFS outcomes. Based on the calibration curves, the nomogram could reliably predict 1-, 3-, and 5-year OS in GBMLGG. KEGG and GO enrichment, GSEA analysis, and gene–gene interaction analysis were all employed to investigate PDCD2's role in GBMLGG. It appears that PDCD2 plays an important role in GBMLGG, as indicated by all these results. Moreover, we found that PDCD2 could significantly promote the proliferation, migration, and invasion of GBMLGG cells.

Now, we have a better understanding of how PDCD2 plays a role in pan-cancer, but there are still some inconsistencies. Despite having explored the relationship between PDCD2 and prognosis in pan-cancer, PDCD2 function has only been confirmed in GBMLGG, but further *in vivo* and *in vitro* studies are needed to elucidate its molecular mechanism in cancer development.

## Conclusions

To sum up, our findings indicate that PDCD2 may play a role in pan-cancer as well as in its prognosis and diagnosis. Decreased PDCD2 expression level was directly related to better survival outcomes of GBMLGG. PDCD2 also could inhibit GBMLGG cell proliferation, migration, and invasion. As a molecular predictor of cancer prognosis, PDCD2 can be used clinically to detect GBMLGG.

## Ethics declaration

This study was authorized by the Institutional Ethics Committees of the First Affiliated Hospital of Chongqing Medical University (approval notice: IACUC-CQMU-2023-0172) and abided by the Declaration of Helsinki.

## Author contributions

TX supervised the manuscript and designed this study. FD, YY, and JH designed this study, performed related assays, and analyzed the data. FD, XC, XZ, LZ, RT, and YZ contributed study materials. All authors read and approved the final version of the manuscript.

## Conflict of interests

The authors declare no competing interests.

## Funding

This study was supported by the 10.13039/501100001809National Natural Science Foundation of China (No. 82172619), the 10.13039/501100005230Natural Science Foundation of Chongqing, China (No. CSTC2021jscx-gksb-N0023), and the Fundamental Research Funds for the Central Universities in China (No. 2022CDJYGRH-002).
